# Stereoselective biotransformation of racemic mandelic acid using immobilized laccase and (S)-mandelate dehydrogenase

**DOI:** 10.1186/s40643-016-0135-3

**Published:** 2017-01-03

**Authors:** Xing Chen, Chengli Yang, Peng Wang, Xuan Zhang, Bingxin Bao, Dali Li, Ruofu Shi

**Affiliations:** Department of Bioengineering, Nanjing University of Science and Technology, Xiaolingwei 200, Nanjing, 210094 People’s Republic of China

**Keywords:** Biocatalysis, (S)-Mandelate dehydrogenase, Laccase, Immobilization, Chitosan, Bi-enzymatic system

## Abstract

**Objectives:**

(S)-Mandelate dehydrogenase (SMDH) and laccase were immobilized on chitosan. The bi-enzymatic system with immobilized SMDH and immobilized laccase was taken to catalyze the stereoselective transformation of racemic mandelic acid and (R)-mandelic acid was obtained from its racemic mixture.

**Results:**

Characteristics of the immobilized enzymes were valuated. The optimum pH and temperature of the immobilized SMDH were found to be pH 3.4 and 45 °C, and these of the immobilized laccase were about pH 6.0 and 55 °C, respectively. The *K*_m_ value of the immobilized SMDH for racemic mandelic acid was 0.27 mM and that of the immobilized laccase for ferrocyanide was 0.99 mM. The thermal and storage stabilities of these enzymes were improved with immobilization. The enantiomeric purity of the bi-enzymatically produced (R)-mandelic acid was determined to be over 99%.

**Conclusion:**

The immobilized bi-enzymatic system for the stereoselective transformation of racemic mandelic acid showed higher productivity, faster reaction velocity, and more stable catalytic ability.

**Graphical abstract:**
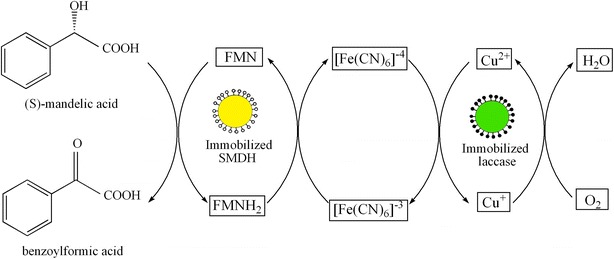
.

## Background

Mandelic acid (MA) was an important drug intermediate and can be large-scalely produced at a relatively low cost by chemical synthesis (Huang and Xu 2006; Lorenz et al. 2002; Tulashie et al. 2010). However, there was an increasing demand for the separation of racemic compound into its chiral constituents in the pharmaceutical and biochemical industries due to the recognition of differences in pharmacological activity of enantiomeric molecules (Mao et al. 2012). (R)-Mandelic acid (R-MA) was a useful chiral material for the production of pharmaceuticals, such as semisynthetic penicillins, cephalosporins, antitumor agents, and antiobesity agents (He et al. 2008; Takahashi et al. 1995). R-MA can be produced by physicochemical methods and biotransformation (He et al. 2008). For example, R-MA can be effectively produced using mandelonitrile as the substrate by the nitrilase (Zhang et al. 2010, 2014). Using racemic mandelic acid as a raw material for the production of R-MA by biocatalysis would be another beneficial and important strategy.

FMN-dependent (S)-mandelate dehydrogenase (SMDH) (EC 1.1.99.31) specially oxidized (S)-mandelic acid (S-MA) to benzoylformic acid (BA) while the cofactor FMN was reduced to FMNH_2_ (Dewanti and Mitra 2003). R-MA can be obtained through stereoselective transformation of racemic mandelic acid, in which S-MA was selectively consumed by SMDH and R-MA was leaved in the reaction system. However, the regeneration of cofactors was usually the main difficulty for the applications of flavin-containing dehydrogenases (Blank et al. 2010). Fortunately, FMN can be regenerated in vitro by laccase when using ferrocyanide as the redox mediator (Baminger et al. 2001). Laccase (EC 1.10.3.2) was a copper-containing phenol oxidase and can oxidate ferrocyanide to ferricyanide, while oxygen was concomitantly reduced to water (Ludwig et al. 2004; Thurston 1994). SMDH and laccase may operate concurrently in one pot to overcome the limitations and disadvantages of a multistep cascade involving reduction and oxidation. In the previous research, a bi-enzymatic system based on coupling SMDH and laccase for the production of R-MA was constructed successfully (Wang et al. 2013). In the bi-enzymatic system, the SMDH catalyzed the oxidation of S-MA to BA and FMN was concomitantly reduced to FMNH_2_, and then FMN was regenerated through the reduction of ferricyanide; the reduced ferricyanide was continuously reoxidized by laccase catalysis. Therefore, the bi-enzymatic system continuously catalyzed the stereoselective transformation of MA and R-MA was obtained from the racemic mixture. Nevertheless, because the free enzymes were put in dialysis bags and were easily inactivated, there was mass transfer limitation in the reaction system, and it limited the further industrial applications of the bi-enzymatic system.

Enzyme immobilization technology was an effective means to benefit the reuse of enzyme and its stability. Immobilization not only enhanced enzyme properties but also facilitated the separation of products. A common method of enzyme immobilization was the covalent linkage of the enzyme to polymeric materials, like chitosan (Silva et al. 2012). Enzyme immobilization on chitosan not only enhanced the chances for reuse but also provided a nontoxic and biocompatible microenvironment conducive to the catalytic activity and stability of the enzyme (Kaur et al. 2014). Like most water-soluble enzymes, the immobilization of SMDH and laccase was a prerequisite for its practical application (Li et al. 2013). In the past three or four decades, immobilization technology has developed rapidly and many kinds of enzymes including laccase have been successfully immobilized on chitosan for biocatalytic reaction (Delanoy et al. 2005; Jiang et al. 2005), and the immobilization of SMDH has also been studied recently and its stability was improved compared to the free counterpart (Wang et al. 2014).

**Fig. 1:**
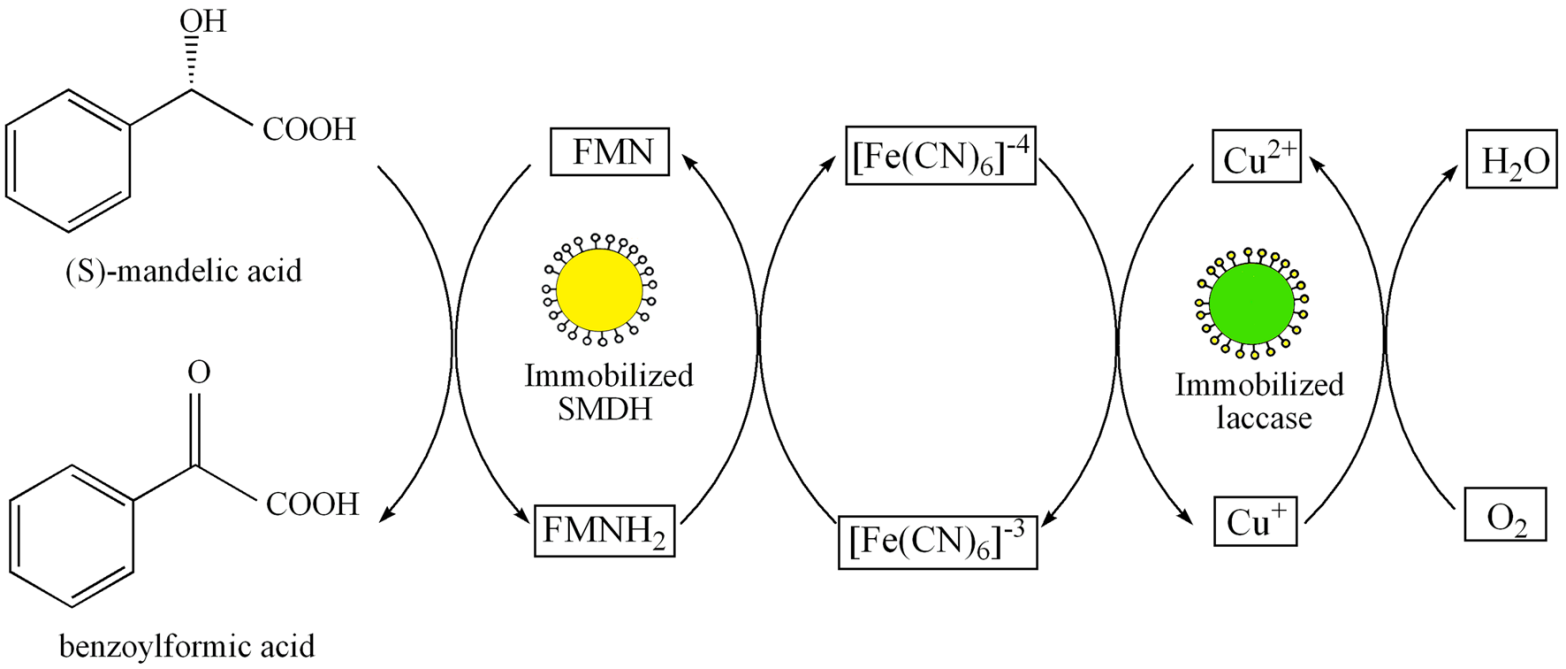
Bi-enzymatic system with immobilized SMDH and immobilized laccase catalyzed continuously the transformation of (S)-mandelic acid to benzoylformic acid using ferricyanide/ferrocyanide as the redox medium

In this study, the SMDH and laccase were immobilized by the strategy that amino groups of enzyme molecules and chitosan were combined through Schiff base linkage by glutaraldehyde. Some enzymatic properties of immobilized SMDH and immobilized laccase, such as optimal pH, optimal temperature, and *K*_m_ value, were studied. In addition, the improved bi-enzymatic system consisting of immobilized SMDH and immobilized laccase catalyzed continuously the stereoselective transformation of MA and R-MA was obtained from its racemic mixture (Fig. 1).

## Methods

### Chemicals

All chemicals used in this study were of the highest grade available and were obtained from Sinopharm Chemical Reagent (Nanjing, China). Toyopearl DEAE-650 M and butyl-Toyopearl 650 M were obtained from Tosoh (Osaka, Japan). Chitopearl BCW-2605 and Chitopearl BCW-2503 (chitosan beads) were purchased from Fujibo (Tokyo, Japan).

### The preparation of SMDH and laccase

The SMDH was produced from the recombinant *Escherichia coli* and the laccase was produced from the fruit bodies of *Agaricus bisporus* as previously described (Wang et al. 2013). The preparation of SMDH used throughout this study was partially purified by a single-anion exchange chromatography step using a Toyopearl DEAE-650 M column with a linear NaCl gradient (0–3.0 M) elution at a flow rate of 1.0 mL/min. On average, 4.9 U/mL of SMDH was produced, corresponding to a specific activity of 20.5 U/mg. The laccase was partially purified by a single-anion exchange chromatography step using a Toyopearl DEAE-650 M column with a linear NaCl gradient (0–3.0 M) elution, and then using a butyl-Toyopearl 650 M column with a linear ammonium sulfate gradient (saturation of 30-0%) elution at a flow rate of 1.0 mL/min. After removing the ammonium sulfate by dialysis, 2.5 U/mL laccase was obtained, corresponding to a specific activity of 2.36 U/mg.

### Enzyme activity assay

The SMDH activity was assayed by measuring the decrease in absorbance of ferricyanide with a U-1800 spectrophotometer at 420 nm as described in the previous study (Wang et al. 2014). One unit of SMDH activity was defined as the amount of enzyme reducing 1 μmol of potassium ferricyanide per minute under the above reaction conditions. The free or immobilized laccase activity was assayed at 30 °C by following the increase in the absorbance of potassium ferricyanide at 420 nm in 200 mM Na_2_HPO_4_-citric acid buffer (pH 6.0) consisting of 20 mM potassium ferrocyanide. One unit of the laccase activity was defined as the amount of enzyme increasing 1 μmol of potassium ferricyanide per minute under the above reaction conditions (Kurokawa et al. 2010).

### Immobilization on chitosan

The chitosan merchandise Chitopearl BCW-2605 was used as the support for the immobilization of SMDH and Chitopearl BCW-2503 was used for immobilization of laccase. 4.0 g of chitosan beads were suspended in 50 mL of 5% (v/v) glutaraldehyde, and the mixture was kept on a rotary shaking incubator for 90 min at 25 °C. Finally, the supports were washed with deionized water to remove unbound glutaraldehyde.

Immobilization of SMDH and laccase were carried out by mixing enzymes with the previously activated supports on a rotary shaking incubator for 90 min at 25 °C. The activated supports were used for immobilization of 20 mL SMDH and 25 mL laccase, respectively. The initial enzyme activity of supernatant was taken as 100%. The immobilization yield (IY) could be calculated according to Eq. (1), where *A* was the initial activity in the supernatant and *B* was the activity of the immobilized enzyme:

1 $$ {\text{IY (\% ) }} = \frac{B}{A} \times 100. $$

### Studies on the enzymatic properties

The effects of pH on free and immobilized laccase activities were performed in 10.0 mL Na_2_HPO_4_-citric acid buffer (200 mM, pH 2.2–8.0) containing 20 mM potassium ferrocyanide at 55 °C. The effects of temperature on free and immobilized laccase activities were performed in 10.0 mL Na_2_HPO_4_-citric acid buffer (200 mM, pH 6.0) containing 20 mM potassium ferrocyanide, and the reaction systems were treated at different temperatures for 15 min before enzymes were put into. The Michaelis constant (*K*_m_) of laccase (immobilized and free) for ferrocyanide was determined using Lineweaver–Burk method at their optimal pH and temperature.

### The thermal stability and storage stability assay

The SMDH-Chitopearl BCW-2605 and laccase-Chitopearl BCW-2503 were separated from the supernatant by filtration, and then were washed with deionized water. Immobilized SMDH and laccase were subjected to thermal stability in Na_2_HPO_4_-citric acid buffer (200 mM, pH 6.0) at their optimal temperatures for 200 h, and were subjected to storage stability in the same buffer at 4 °C. The control experiment was performed with the free enzymes. Samples were withdrawn periodically and their residual activities were measured to evaluate the thermal stability and storage stability. The initial activity was taken as 100%.

### The bi-enzymatic system consisting of immobilized SMDH and immobilized laccase catalyzed the stereoselective transformation of mandelic acid

The stereoselective transformation of MA was aerobically catalyzed by the coupling of immobilized SMDH and immobilized laccase with ferricyanide or ferrocyanide as the redox mediator. The experiment was performed at 30 °C and was oxygenated by incubating on a rotary shaking incubator (140 rpm) in 150 mL beakers. The reaction system contained 10 mM potassium ferrocyanide, 1.0 g immobilized SMDH, 1.0 g immobilized laccase, and MA with concentrations of 10, 20, 30, 40, 50, and 60 mM in 50 mL Na_2_HPO_4_-citric acid buffer (200 mM, pH 6.0). At fixed intervals, 10 μL, the reaction mixture was withdrawn to monitor the course of the reaction by high-performance liquid chromatography (HPLC), which has been described in our previous description (Wang et al. 2014, 2013). When the concentration of MA was optimized in the reaction system, the stereoselective transformation and reuse of the bi-enzymatic system were conducted under the same conditions.

### Analytical methods

MA and BA were analyzed by HPLC using a C_18_ column (VP-ODS, 150 mm × 4.6 mm, Shimadzu, Japan) at 25 °C. They were detected at 220 nm with a Shimadzu SPD-10A detector. The mobile phase consisted of methanol and phosphate buffer (6.6 g/L Na_2_HPO_4_, 6.8 g/L KH_2_PO_4_) (1:9, v/v), and the flow rate was 1.0 mL/min.

The (S)-mandelic acid and the (R)-mandelic acid were analyzed by HPLC using a chiral column (γ-CD, 150 mm × 4.6 mm, YMC, Japan) at 25 °C. They were detected at 254 nm with a Shimadzu SPD-10A detector. The mobile phase consisted of phosphate buffer (6.6 g/L Na_2_HPO_4_, 6.8 g/L KH_2_PO_4_), ethanol, and acetonitrile (65:20:15, v/v), and the flow rate was 1.0 mL/min.

## Results and discussion

### Immobilization

**Fig. 2:**
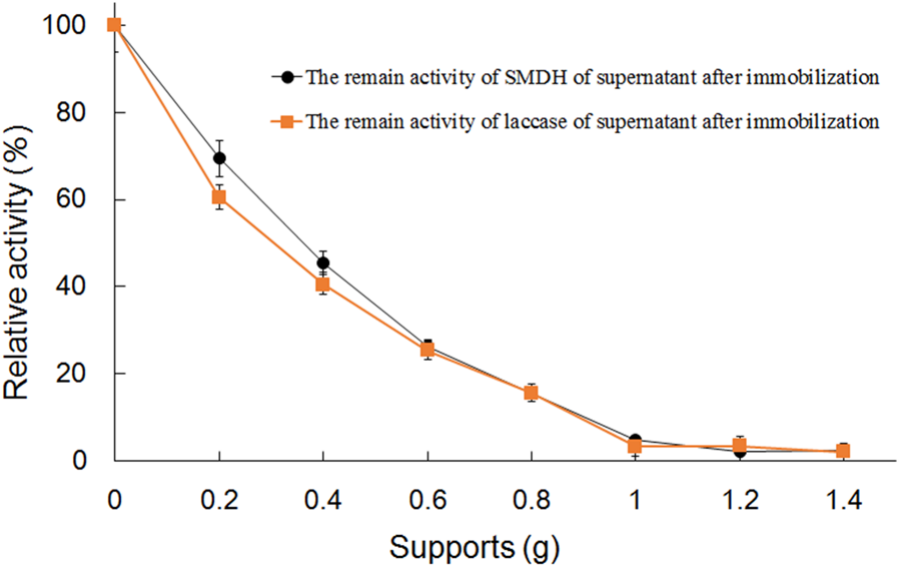
Immobilization of SMDH and laccase on glutaraldehyde-activated chitosan beads. The initial enzyme activity of supernatant was taken as 100%. Reactions were performed in triplicate, and* error bars* represented standard deviations (StDev)

The immobilization of SMDH and laccase on glutaraldehyde-activated chitosan beads was studied. The results were shown at Fig. 2. It was found that 1.2 g support was the optimal support content for both 5.0 mL SMDH and laccase immobilization. After calculation, the immobilization yield for SMDH was 62.69% and that for laccase was 62.4%; the activity of immobilized SMDH was 12.8 U/g and that of immobilized laccase was 6.5 U/g. The behavior of high immobilization yield and immobilized enzyme activity may be explained by the fact that the strategy of immobilization was appropriate and the amine groups in chitosan have been highly activated with glutaraldehyde, and amine groups of the proteins were easy to link with glutaraldehyde-activated chitosan.

### Enzymatic properties

**Fig. 3:**
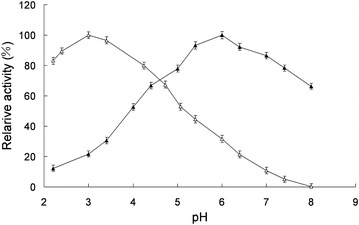
Effects of pH on free and immobilized laccase activities. *Open triangle* indicated soluble laccase, and *close triangle* indicated immobilized laccase. Reactions were performed in triplicate, and* error bars* represented standard deviations (StDev)

The effects of pH on the activity of free and immobilized laccase for the oxidation of ferrocyanide were studied, and the results were presented in Fig. 3. The free laccase showed an optimum pH of 3.0 and the optimum pH of immobilized laccase was 6.0, and the immobilized enzyme had a broad pH range of high activity. The microenvironment of the immobilized enzyme was affected by the properties of the carrier, resulting in differences between the free and immobilized enzyme (Gupta and Kumar 2000).

**Fig. 4:**
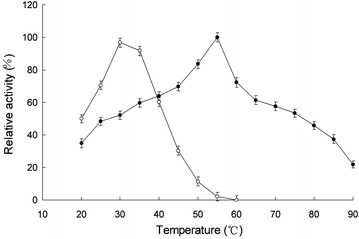
Effects of temperature on free and immobilized laccase activities. *Open circle* indicated soluble laccase, and *close circle* indicated immobilized laccase. Reactions were performed in triplicate, and *error bars* represented standard deviations (StDev)

The temperature dependence of the activities of the free and immobilized laccase were studied in Na_2_HPO_4_-citric acid buffer (200 mM, pH 6.0) in the temperature range 20–90 °C and temperature profiles of the free and immobilized laccase were shown in Fig. 4. It showed that the immobilized laccase had a broad temperature range of high activity. Optimum temperature was found at about 30 °C for free laccase and 55 °C for immobilized laccase. The support had a protecting effect at high temperatures at which enzyme deactivation took place. The conformational flexibility of the enzyme was affected by immobilization. Immobilization of laccase on chitosan beads caused an increase in the enzyme rigidity which was commonly reflected by increase in stability towards denaturation by raising the temperature (Jiang and Zhang 1993; Mohamed and Naby 1993).

The *K*_m_ of immobilized laccase for ferrocyanide was about 0.99 mM, which was greater than that of free one (0.12 mM). An increase in *K*_m_ for an immobilized enzyme indicated that the immobilized enzyme had an apparent lower affinity for its substrate than that of the free enzyme, which may be caused by the steric hindrance of the active site by the support, the loss of enzyme flexibility necessary for substrate binding, or diffusional resistance to solute transport near the particles of the support (Çetinus and Öztop 2003).

Recently, we have shown the enzymatic properties of immobilized SMDH (Wang et al. 2014), and the immobilized SMDH showed maximum activity at pH 3.4 and 45 °C and its *K*_m_ value was 0.27 mM. In addition, the *K*_m_ value of the immobilized SMDH was lower than the free SMDH, and it indicated that immobilized SMDH had an apparently higher affinity for its substrate than that of the free enzyme.

### Effects on the thermal stability and storage stability

**Fig. 5:**
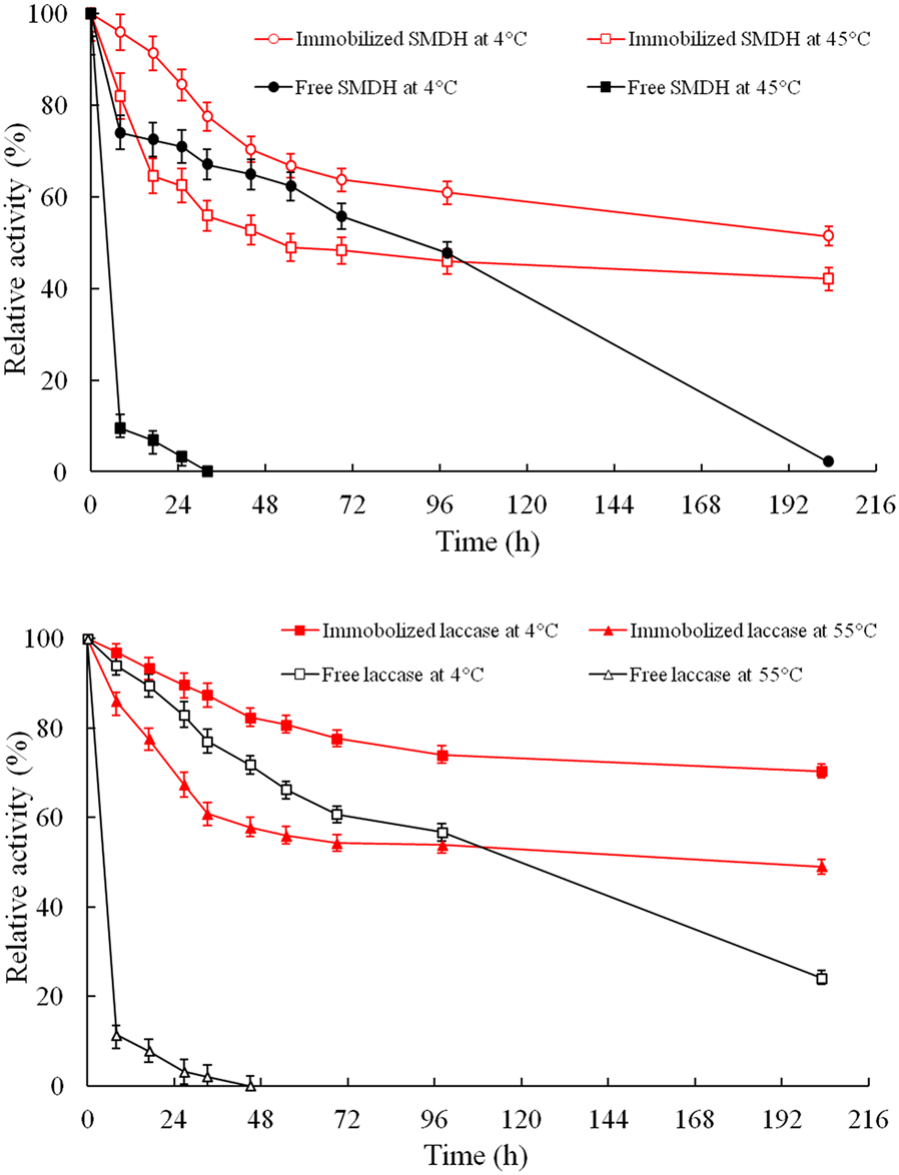
Change of thermal stability and storage stability of immobilized SMDH and immobilized laccase over time. Reactions were performed in triplicate, and *error bars* represented standard deviations (StDev)

Figure 5 showed the changes of thermal deactivation and storage stability of immobilized enzymes (SMDH and laccase). When incubation at their optimal temperatures for 8 h, immobilization reduced the deactivation rate of SMDH about fivefold and that of laccase about ninefold, which suggested that the thermostability of the immobilized enzymes was significantly higher than that of the free enzymes at their optimal temperature. When storage at 4 °C for 200 h, there was about 1.4-fold reduction of deactivation rate for immobilized SMDH and about threefold for immobilized laccase, so it showed immobilized enzymes had improved storage stability. The decrease in activity was explained as a time-dependent natural loss in enzyme activity, and this was prevented to a significant degree by immobilization (Lemainque et al. 1988). The residual enzyme activity of immobilized SMDH was about 40% and that of immobilized laccase was 55% at their optimal temperatures for 200 h. The residual enzyme activity of immobilized SMDH was about 60% and that of immobilized laccase was 80% after storage at 4 °C for 200 h. In addition, the thermal stability and storage stability of the immobilized laccase were higher than the immobilized SMDH.

The high stability of immobilized enzymes at high temperatures represented that the support had a protecting effect for SMDH and laccase. When enzyme was in its free form, it presented some flexibility, which meant that its active site underwent irreversible conformational changes, causing inactivity. When it was immobilized, a more rigid form was acquired because of covalent linkages to the support. Enzymes’ rigidification may lead to preservation of the enzyme properties under drastic conditions (Rodrigues et al. 2013). This stiffness decreased the enzyme’s flexibility, maintaining the form of the active site, which was responsible for its activity (Silva et al. 2012).

### Biotransformation experiments with immobilized enzymes catalyzing

The SMDH specially catalyzed the oxidation of S-MA to BA, while its cofactor FMN was reduced, then the reduced FMN was regenerated by ferricyanide which was regenerated by laccase catalyzing the oxidation of ferrocyanide. Therefore, the coupling of SMDH and laccase can oxidize continuously S-MA to BA. To improve stability of the biocatalysts and reuse them easily, free SMDH and laccase were immobilized on chitosan beads.

**Fig. 6:**
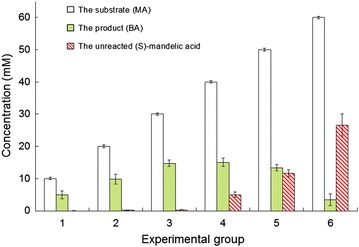
Results of biotransformation reaction using different concentration of MA as the substrate catalyzed by the improved bi-enzymatic system after 14 h. The reaction system contained different concentration of MA, 10 mM potassium ferrocyanide, 1.0 g immobilized SMDH, 1.0 g immobilized laccase, and 50 mL Na_2_HPO_4_-citric acid buffer (200 mM, pH 6.0). Reactions were performed in triplicate, and *error bars* represented standard deviations (StDev)

A bi-enzymatic system consisting of free SMDH and laccase effectively catalyzed the oxidation of S-MA using a small number of redox medium and R-MA was obtained from its racemic mixture, which has been reported in recent study (Wang et al. 2013). Compared with the free bi-enzymatic system, the immobilized bi-enzymatic system showed obvious advantages, such as higher productivity, faster reaction velocity, and more stable catalytic ability. Comparison of the catalytic performance of the immobilized and free bi-enzymatic system was conducted (Table 1). The result of the free bi-enzyme system was reported in the recent study (Wang et al. 2013). As it was shown in Table 1, the handling capacity of substrate concentration increased threefold and the reaction velocity grew from 0.85 μmol/h per U of SMDH to 11.6 μmol/h per U of SMDH, which may owe to the improved stability of immobilized enzymes and the increase of mass transfer rate. However, the bi-enzymatic system may be inhibited by its substrate, leading to the transformation rate rising to a maximum and then descending as the substrate concentration increase, as shown in Fig. 6. Substrate inhibition was an extremely widespread phenomenon in enzyme kinetics (Reed et al. 2010). Further study will focus on the mechanism of substrate inhibition. To maximize the yield of R-MA, the reaction conditions needs to be further optimized by a kinetic model describing S-MA conversion in the bi-enzymatic system.

**Table 1:** Differences of the bi-enzymatic system with immobilized enzymes and free enzymes

The bi-enzymatic system	Total activity of SMDH (U)	Total activity of laccase (U)	Substrate concentration (mM)	Reaction time (h)	Reaction velocity (μmol/h∙U)
The free bi-enzymatic system	49.0	25.0	10.0	12.0	0.85
The immobilized bi-enzymatic system	12.9	6.50	30.0	10.0	11.6

**Fig. 7:**
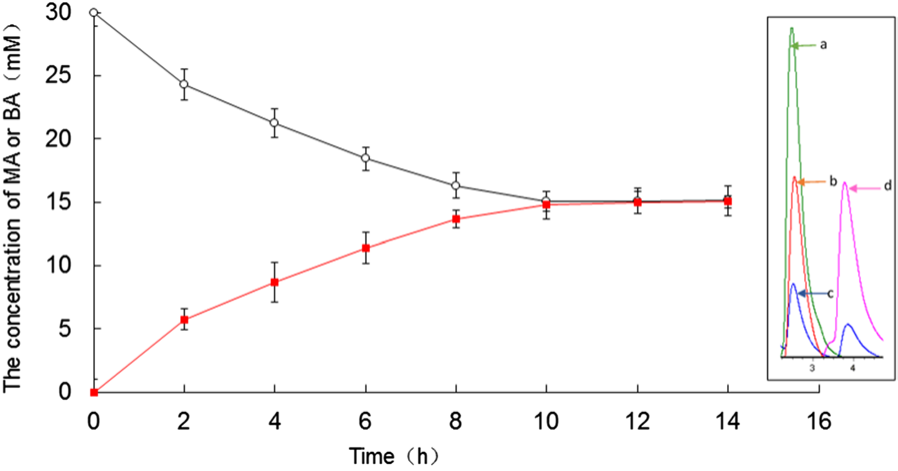
Bi-enzymatic system consisting of immobilized SMDH and immobilized laccase catalyzed the conversion of (S)-mandelic acid to benzoylformic acid. *Open circle* indicated MA and *close square* indicated BA, respectively. Reactions were performed in triplicate, and *error bars* represented standard deviations (StDev). *Inset* HPLC trace of MA and BA at the beginning and the end of stereoselective transformation. **a** HPLC of MA; **b** HPLC of the beginning reaction mixture; **c** HPLC of the end reaction mixture; **d** HPLC of BA

**Fig. 8:**
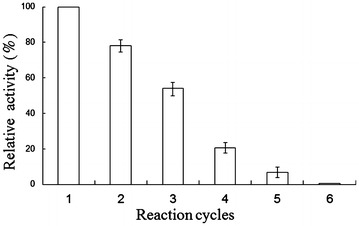
Reusability of the bi-enzymatic system consisting of immobilized SMDH and immobilized laccase. Reactions were performed in triplicate, and *error bars* represented standard deviations (StDev)

Figure 7 showed the transformation process of S-MA to BA using 30 mM MA, which was catalyzed by the bi-enzymatic system consisting of immobilized SMDH and immobilized laccase. Analyzing Fig. 7, it was clear that S-MA was continuously oxidized to BA, and the transformation was close to complete within 10 h. The reaction mixture after 10 h reaction was analyzed by HPLC, and it was found that there was no products other than BA and R-MA. The enantiomeric purity of the bi-enzymatically produced MA could be determined to be over 99% for the (R)-enantiomer by chiral HPLC. Figure 8 showed the enzyme reuse of the bi-enzymatic system. The enzyme activity declined gradually with more cycles. The bi-enzymatic system retained its 80% of the original activity after one cycle, while it no longer showed catalytic activity after five cycles. Due to the weak interaction of Schiff base linkage between the enzymes and supports, the enzymes can release itself from the immobilized enzymes. However, the bi-enzymatic system still had advantages in reuse over the free enzyme.

Although the bi-enzymatic system with immobilized enzymes for the stereoselective transformation of MA was constructed, there were several challenges. Further study, more parameters would be optimized, such as the effect of ferrocyanide, support and immobilization method, and the co-immobilization of SMDH and laccase would be done.

## Conclusions

SMDH and laccase have been successfully immobilized on chitosan. The thermal stability and storage stability of SMDH and laccase were improved by immobilization, where it was likely that immobilization prevented hydrophobic patches from aggregating. The improved properties may indicate applicability of the immobilized SMDH and laccase for the continuous bi-enzymatic reaction. Using the bi-enzymatic system with immobilized enzymes for the stereoselective transformation of MA, it showed higher productivity and faster reaction velocity than the free enzymes did. Besides, the purity of the bi-enzymatically produced R-MA exceeded 99% using chiral HPLC.
